# Every Body Has Value: Ending Discriminatory Restrictions on Anatomical Donations

**DOI:** 10.1093/infdis/jiaf413

**Published:** 2026-03-17

**Authors:** Sara Gianella, Jeff Taylor, Brittany Shelton, Nina Martinez, Robert Deiss, Karine Dubé, Maile Karris

**Affiliations:** 1 Department of Medicine, University of California, San Diego, San Diego, California, USA; 2 HIV+Aging Research Project–Palm Springs, Palm Springs, California, USA; 3 Department of Public Health, University of Tennessee, Knoxville, Knoxville, Tennessee, USA; 4 Independent Researcher, Atlanta, Georgia, USA

**Keywords:** HIV, body donation, donor eligibility, stigma, end-of-life research

## Abstract

Anatomical body donation plays a critical role in medical education and scientific discovery. Yet, most programs in the United States continue to exclude individuals with human immunodeficiency virus (HIV) or viral hepatitis, despite modern biosafety protocols and decades of scientific progress. These outdated restrictions are rooted in historical stigma rather than current risk, and they unjustly deny people living with these conditions the opportunity to make a final, meaningful contribution to science. In this Viewpoint, we call for the urgent revision of donor eligibility policies to reflect contemporary understanding of infectious disease transmission and universal precautions. Drawing on examples from end-of-life HIV research and the broader transplant landscape, we argue that inclusion is both scientifically sound and ethically imperative. Every person deserves the dignity of being valued in death as in life, and no body should be excluded based on fear, misinformation, or outdated policy.

The donation of human bodies for anatomical study is a cornerstone of medical education, offering students and researchers a vital opportunity to understand the complexity of the human form. These donations not only cultivate anatomical knowledge but foster empathy, teamwork, and respect, qualities essential to compassionate medical care. Academic medical institutions across the world depend on body donation programs to train the next generation and advance science [1].

Unfortunately, despite decades of progress in bioethics, prevention, and treatment, human immunodeficiency virus (HIV) and hepatitis B and C remain the most common exclusion criteria for body donation, as cited by >80% of body donation programs [2]. These restrictions are rooted more in legacies of arcane stigma and misplaced fear than scientific understanding. The American Association for Anatomy calls on institutions to modernize their policies, urging them to eliminate unnecessary exclusions that disregard current biosafety standards and perpetuate discrimination. They recommend involving infectious disease experts in the development and review of donor eligibility criteria to update current policies [1].

## A LEGACY OF STIGMA AND OBSOLETE PRACTICES

Exclusionary policies trace back to the early HIV epidemic, a time of widespread fear and misinformation [3]. Universal precautions were not widely implemented, and HIV was often fatal when it progressed to AIDS. In that context, such restrictions were reasonable and justifiable. But in 2025, we benefit from decades of scientific progress including effective and tolerable preventive medication, treatment as prevention (colloquially known as “U = U”), and postexposure prophylaxis (PEP) of HIV. Highly protective hepatitis A and B vaccines, effective treatments for hepatitis B, and a cure for hepatitis C also exist.

Standard biosafety protocols for handling donated biological materials, including gloves, gowns, face shields, and rigorous sterilization, provide effective protection for students and staff [4-6]. Since 1991, the Occupational Safety and Health Administration has mandated that human biological materials be treated as potentially infectious, as bodies are not routinely screened for communicable diseases. Knowing that a donor acquired HIV or chronic viral hepatitis further heightens safety awareness and reinforces best practices. Routine vaccination for common infections including hepatitis A and B, combined with universal precautions, and occupational HIV PEP render donor exclusions based on HIV and hepatitis status scientifically unjustified.

These policies are not just anachronistic, they also perpetuate harmful messages: that people with HIV and/or hepatitis are less worthy, their bodies less valuable, their deaths more dangerous. On 26 June 2025 [7], the Organ Procurement and Transplantation Network made kidney and liver transplantation between people with HIV standard clinical care; transplants of organs other than liver and kidney remain permitted under clinical research protocols per the HIV Organ Policy Equity Act of 2013. People with HIV are now routinely being encouraged to consider donating life-saving organs, and they should be able to make the final, generous decision of donating their bodies to science.

## A SCIENTIFIC AND ETHICAL LOSS

**Figure 1.**
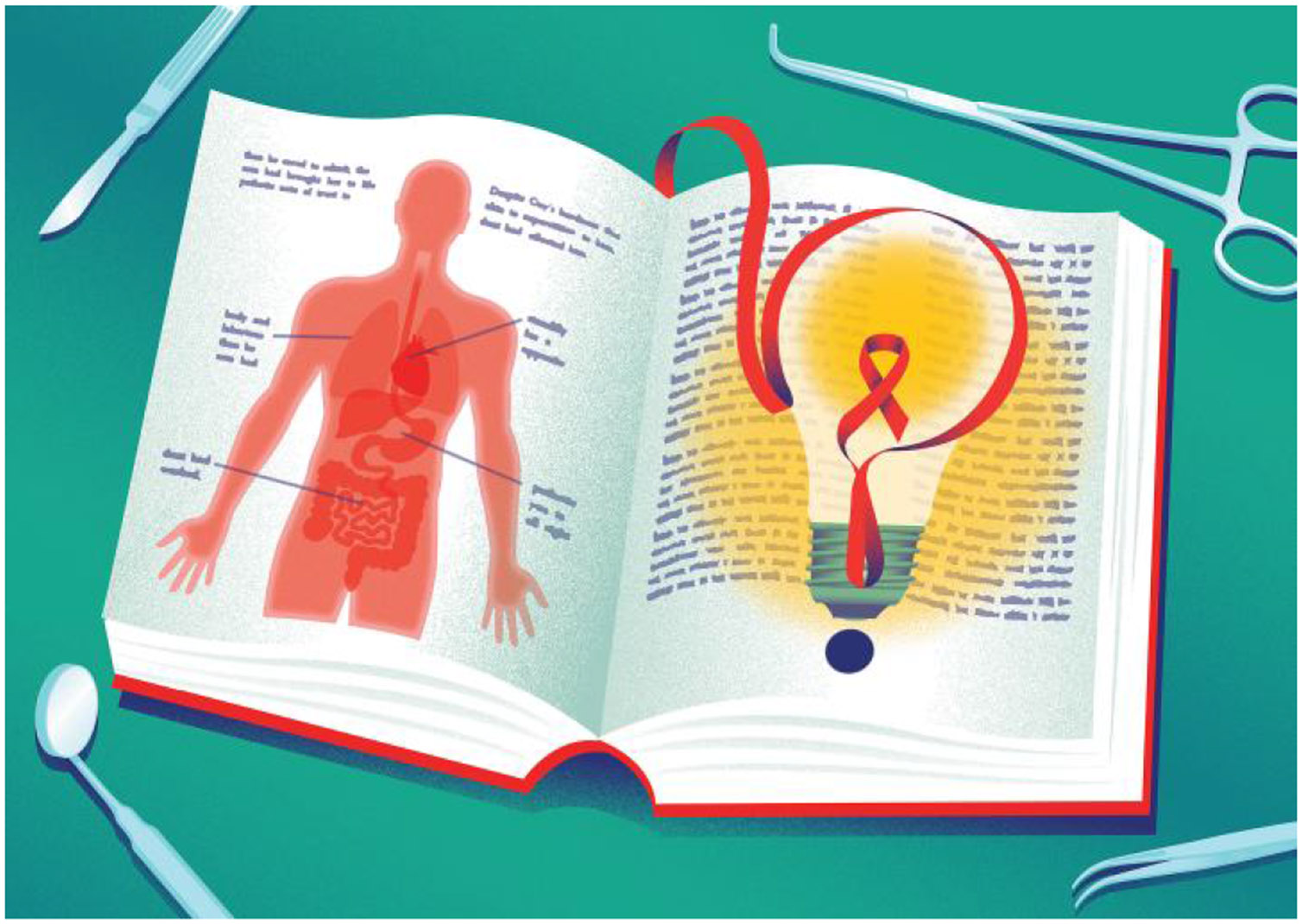
This artwork is a symbolic representation of the scientific value of body donation by people with HIV or hepatitis. The open book and light bulb represent the contributions to knowledge and discovery, while the red ribbon honors individuals living with or affected by HIV. It serves as a reminder that every body has value.

These exclusions are not only discriminatory but also contribute to loss of scientific and medical knowledge (Figure 1). Programs like the Last Gift study demonstrate the profound value of end-of-life research in this populations [8]. Through the generosity of participants, we are advancing our understanding of HIV reservoirs and viral persistence. For example, we confirmed the presence of replication-competent virus in the brains of people on suppressive antiretroviral therapy [9], observed migration of infected cells across tissues [10], and identified proviruses in deep tissue compartments that may resist curative strategies [11]. But the impact of this work goes beyond scientific discovery. For many Last Gift participants, body donation is an act of agency, dignity, and hope: a way to leave a legacy that helps others and reclaim a narrative too often shaped by stigma and exclusion [12].

## THIS IS NOT JUST A POLICY QUESTION, IT’S A HUMAN PRINCIPLE

People with HIV have endured decades of exclusion. They have faced discrimination in healthcare, employment, housing, and beyond. Entire communities were lost to a pandemic that was ignored for too long. Despite that history, many still wish to give back, and to deny them at life’s end is a betrayal. It denies them autonomy, robs them of agency, and sends one final, cruel message: that their lives, and their deaths, do not matter. It is long past time to retire these obsolete restrictions.

## CALL TO ACTION

All institutions with body donation programs should review and revise their infectious diseases eligibility criteria. Policies should reflect current biosafety practices (including universal vaccination and rapid access to PEP), prioritize inclusion, and recognize the value of every person, every body.

Medical schools and research institutions must educate their students, staff, and faculty about the importance of just policies. We must celebrate the courage and generosity of those who choose to make this final gift, especially those from communities historically marginalized in science and medicine.

Finally, we must center the voices of people with HIV and others living with stigmatizing conditions; their lived experiences, insights, and dignity must guide the way forward. Every body has something to teach, and no one should be denied the chance to leave behind a legacy of knowledge for outdated reasons.
